# Bright’s Disease, Etc.

**Published:** 1886-11

**Authors:** 


### Book Reviews.

Bright's Disease and Allied Affections of the Kidneys. *By* Charles W. Purdy, M.D., *Queen's University, Professor of Genito-Urinary and Renal Diseases in the Chicago Policlinic, etc. 8vo, pp, 288, with 18 illustrations. Philadelphia:* Lea Brothers & Co., *1886. Chicago:* A. C. McClurg & Co.

This work is nicely gotten up; is printed upon good paper, in clear, handsome type, and for a first edition is remarkably free from typographical errors.

We believe it is the author's first venture into the realm of book-making, although he has been a frequent contributor to medical journals. We congratulate Dr. Purdy, first, because he writes such clear, terse, straightforward

English, and, secondly, because he has succeeded in writing so excellent a work on "Bright's Disease and Allied Affections of the Kidneys." The work is divided into nine (9 chapters, as follows: (1) Albuminuria: (2) Uræmia: (3) Acute Nephritis: (4) Chronic Nephritis: (5) Cirrhosis of the Kidneys: (6) Scarlatinal Nephritis: (7) Puerperal Nephritis: (8) Lardaceous Degeneration of the Kidneys, and (9) Cyanotic Induration of the kidneys.

In the first chapter we find a very clear statement of the present doctrines regarding albuminuria, together with explicit directions as to the modes of testing for albumen. The chapter closes with advice on the hygienic treatment of albuminuria, which we have read with pleasure and profit, but the subject of "Renal Casts," which is discussed in these chapters—but why, we can hardly see—receives, according to our way of thinking, very inadequate treatment. In a little more than two pages the author condenses all he has to say concerning renal casts. It is undeniably true that the tube-cast is capable of imparting more and more accurate information as to the actual state of the renal tubules in Bright's disease than can be obtained in any other way. Hence the vast importance of the careful study. Hence, also, the necessity of extended descriptions of their varied phases in our text-books. We are, therefore, fully warranted in entering a good-natured protest against Dr. Purdy's book, in which he discusses so important a subject in a manner so unsatisfactory.

The chapter on "Uræmia" is excellent from first to last. For the general practitioner it is one of the best articles on uræmia that we have ever seen. We do not subscribe very heartily to the author's doctrine of giving "subcarbonate of iron in large doses—twenty to thirty grains—and repeated every three hours," as a means of "preventing or lessening the intensity of the convulsions," nor do we consent by any means to allow New York to claim the credit of having "demonstrated" that morphia can be used hypodermically or otherwise in anæmic convulsions, since we have so used it for ten years past, and always in response to sound therapeutic indications.

The two following chapters on "Acute and Chronic Nephritis," respectively, are quite up to the times in all regards, and are really models of clear, concise composition. They are admirable reading for the young practitioner who encounters his first case of acute or chronic Bright's disease without much clinical experience, since they are characterized by clear descriptions of the diagnostic phenomena as well as definite and well considered rules of therapeutic procedure. We would modify the term "Nephritis" by some title less general and more definite, as "Tubal," or "Parenchymatous" Nephritis. On physiological grounds we think strong objections can be urged against the practice urged by Dr. Purdy and others, of "rendering the urine alkaline and maintaining it so by the administration of a saline," for the purpose of dissolving renal casts, which obstruct the tubules, but we have not time or space to consider the subject now.

Perhaps the crowning chapter in the book is the one on "Scarlatinal Nephritis," which may be ranked as a classic article on this formidable disease. We should like to quote it entire, but being unable to do that, we must be content with saying that it is justly entitled to great praise as a careful, well-considered and well-written article upon a most important and interesting subject. Finally, we commend the book to the profession as an admirable treatise on an important class of diseases, which are too little understood by the general practitioner. We have pointed out a few, and we think all, of its faults; its excellences are so many that we cannot even enumerate them. It is not only a Chicago book, but it is the work of a personal friend, and as such we extend to it a hearty welcome. I. N. D.

